# Will Employee–AI Collaboration Enhance Employees’ Proactive Behavior? A Study Based on the Conservation of Resources Theory

**DOI:** 10.3390/bs15050648

**Published:** 2025-05-09

**Authors:** Chenxi Sun, Xinan Zhao, Baorong Guo, Ningning Chen

**Affiliations:** 1 School of Business Administration, Northeastern University, Shenyang 110167, China; 1710445@stu.neu.edu.cn (C.S.); xnzhao@mail.neu.edu.cn (X.Z.); 1810446@stu.neu.edu.cn (N.C.); 2 Business School, Guilin University of Technology, Guilin 541006, China

**Keywords:** employee–AI collaboration, proactive behavior, workload, AI literacy

## Abstract

This study explores how employee–AI collaboration can promote employees’ proactive behavior by reducing their workload, and examines the mediating role of workload and the moderating effect of AI literacy. Based on a survey of employees across multiple industries, the study finds that employee–AI collaboration significantly reduces employees’ workload, which in turn encourages more proactive behavior. In this process, workload serves as a central mediating mechanism, as it helps alleviate task pressure and frees up cognitive resources, enabling employees to take on additional responsibilities and put forward innovative suggestions. Furthermore, with increasing levels of employee–AI collaboration, employees with higher AI literacy tend to experience greater workload relief, while those with lower literacy demonstrate a stronger and more consistent proactive behavioral response. These findings offer theoretical insight into employee–AI interaction and practical implications for enhancing initiative and innovation through effective AI integration.

## 1. Introduction

In today’s digitally driven organizations, employee–AI collaboration has become a strategic lever for enhancing productivity and advancing digital transformation (Marvi et al., 2025; D. Wang et al., 2020). It refers to the process through which employees and AI systems jointly engage in task execution, with AI functioning not merely as a tool, but as a cognitive or operational partner. As artificial intelligence (AI), robotics, and big data analytics evolve, organizations are increasingly deploying intelligent systems to augment human effort (Park et al., 2019; Wu et al., 2024). These partnerships support improvements in operational efficiency, facilitate the handling of complex tasks, and stimulate innovation across various sectors (Cabrera et al., 2023). For example, AI-powered robots enhance precision in manufacturing, automated platforms improve customer interactions, and data-driven systems assist in informed decision-making within knowledge-intensive work. In this context, employee–AI collaboration is emerging as a foundational element of contemporary organizational practice (Wu et al., 2025).

While such collaboration is transforming work processes, its implications for individual employee behavior remain insufficiently understood (Reverberi et al., 2022). Existing studies suggest that AI can relieve employees from repetitive or low-value tasks, enabling them to redirect their efforts toward more cognitively demanding and meaningful activities (Li et al., 2023; Wu & Zhang, 2024). A particularly important behavioral outcome in this setting is proactive behavior—defined as self-initiated, future-oriented actions intended to improve personal or organizational performance (McCormick et al., 2019). These behaviors encompass innovation, problem-solving, and constructive voice, all of which are critical to maintaining competitive advantage (Bjørkelo et al., 2010). However, most research has emphasized outcomes such as job satisfaction or task performance, offering limited insight into how employee–AI collaboration specifically shapes proactive behavior (Li et al., 2024a). Moreover, the underlying mechanisms, especially regarding how AI-related task shifts alter employee engagement and resource use, have yet to be fully theorized. Addressing this gap is essential, as proactivity underpins innovation, technological adaptation, and long-term organizational resilience. As AI continues to redefine work, understanding how it enhances rather than displaces human agency becomes an urgent priority.

To examine this question, this study draws upon Conservation of Resources (COR) theory, which holds that individuals seek to acquire, retain, and protect resources while minimizing loss (Hobfoll & Shirom, 2000). In this framework, workload is viewed as a key resource-depleting factor, encompassing physical, cognitive, and emotional effort at work (Gopher & Donchin, 1986). Through collaboration with AI, employees may experience relief from repetitive or high-intensity tasks, allowing for the preservation and reallocation of energy toward higher-order activities (Yin et al., 2024). Reducing workload in this way not only provides more time and mental space, but also supports autonomy and reduces stress—conditions conducive to proactive behavior (Calzarossa et al., 2016). Accordingly, this study positions workload reduction as the primary pathway through which employee–AI collaboration fosters proactivity.

At the same time, employees differ in their capacity to engage effectively with AI systems. AI literacy, defined as an individual’s ability to understand, operate, and apply AI technologies, may shape how collaboration impacts workload and behavior (Su et al., 2023). Employees with high AI literacy are often more confident, technically adept, and receptive to integrating AI into their work routines, enabling greater gains in efficiency and innovation (Ng et al., 2021). In contrast, those with limited AI literacy may struggle to adapt, viewing AI as a threat to competence or job security—perceptions that can dampen initiative and hinder behavioral engagement (Chiu et al., 2024; Zhou et al., 2025). Thus, AI literacy may moderate the relationship between employee–AI collaboration and proactive behavior by influencing both perceived workload and the psychological response to AI-driven change.

In summary, drawing on Conservation of Resources theory, this study explores how employee–AI collaboration promotes employees’ proactive behavior through optimized resource allocation, and further examines the moderating role of AI literacy. By articulating the mechanisms involved, the study contributes to a more nuanced understanding of employee–AI interaction and provides actionable insight for organizations seeking to integrate intelligent systems while sustaining human initiative and innovation.

## 2. Theoretical Foundation and Research Hypotheses

### 2.1. Employee–AI Collaboration and Proactive Behavior

According to Conservation of Resources (COR) theory, individuals are intrinsically motivated to acquire, protect, and expand their valued resources. When employees perceive that their resources are preserved or enhanced, they experience a greater sense of psychological security and control, which encourages proactive engagement at work (Hobfoll & Shirom, 2000). As an innovative work arrangement, employee–AI collaboration reshapes employees’ resource dynamics by reducing workload and alleviating repetitive, high-intensity tasks (Marvi et al., 2025). AI technologies can automate routine functions and support decision-making processes, enabling employees to redirect their attention toward cognitively demanding and creative tasks (Vardanyan, 2022).

This redistribution of effort reduces physical and mental fatigue and provides the psychological space for innovation, initiative, and task improvement. It also enhances positive psychological states, such as self-efficacy, confidence, and autonomy, which further motivates employees to contribute constructively to organizational goals (Hornberger et al., 2023; Bai et al., 2025). For example, they may proactively propose new ideas, coordinate team efforts, or assume additional responsibilities to improve collective performance (Li et al., 2023).

Beyond immediate stress relief, COR theory emphasizes the role of resource gain in sustaining motivation. When individuals perceive their resources to be increasing, they are more likely to invest in further resource development and engage in discretionary behaviors that create long-term advantages (Hobfoll & Shirom, 2000; Fryer & Payne, 1984). By fostering resource conservation and promoting constructive psychological states, employee–AI collaboration facilitates this gain cycle, strengthening work motivation and behavioral investment (Vardanyan, 2022). It also supports employee growth by enabling upskilling and increasing their willingness to take on complex challenges (Fan & Smith, 2017). In contexts of organizational change or uncertainty, employees with such support may demonstrate greater adaptability and propose effective responses (Sundar, 2020).

These mechanisms indicate that employee–AI collaboration enhances proactive behavior through multiple pathways, including workload relief, positive affective activation, and reinforcement of the resource gain process (Salanova & Schaufeli, 2008). Therefore, the following hypothesis is proposed:

**H1:**  *Employee–AI collaboration positively influences employees’ proactive behavior.*

### 2.2. The Mediating Role of Workload

In line with Conservation of Resources (COR) theory, individuals strive to preserve and accumulate valuable resources such as time, energy, and cognitive capacity, while minimizing their depletion (Hobfoll & Shirom, 2000). Employee–AI collaboration contributes to this preservation by automating low-value and repetitive tasks, thereby reducing employees’ workload (Li et al., 2024a; Marvi et al., 2025). For instance, AI tools such as chatbots for customer service or analytics platforms for data processing can manage routine responsibilities like information retrieval and pattern recognition, thereby alleviating the time and effort required of employees (Vardanyan, 2022; Zheng et al., 2022). As a result, employees experience less physical and mental strain and can shift their attention toward more meaningful and cognitively demanding work, which in turn supports their psychological well-being and job satisfaction (Timperley & Robinson, 2000; Guingrich & Graziano, 2024). Therefore, the following hypothesis is proposed:

**H2:**  *Employee–AI collaboration negatively influences employees’ workload.*

Beyond its direct health benefits, reduced workload enables employees to reinvest cognitive and emotional resources into tasks that require higher engagement and creativity. COR theory suggests that such resource restoration fosters positive psychological states, particularly autonomy, competence, and motivation, which serve as precursors to proactive behavior (Hobfoll & Shirom, 2000). When workload is lightened, employees are more likely to initiate improvements, engage in complex problem-solving, and contribute to innovation (Gaba & Lee, 1990). Empirical research further indicates that reduced strain diminishes burnout and emotional exhaustion, thereby increasing employees’ psychological capacity to act proactively (Sperandio, 1971; Bowling et al., 2015). In contrast, excessive workload, especially in cognitively demanding roles, can limit the mental bandwidth necessary for exploring new opportunities and hinder proactive engagement (Kosch et al., 2023). By easing these constraints, workload reduction expands employees’ capacity for forward-looking, self-initiated contributions (Salanova & Schaufeli, 2008). Therefore, the following hypothesis is proposed:

**H3:**  *Employees’ workload negatively influences employees’ proactive behavior.*

Collectively, these findings indicate that workload functions as a mediating mechanism in the relationship between employee–AI collaboration and proactive behavior. As repetitive or resource-draining tasks are offloaded to AI systems, employees regain the cognitive and emotional bandwidth needed for initiative, innovation, and organizational participation. Therefore, the following hypothesis is proposed:

**H4:**  *Workload mediates the relationship between employee–AI collaboration and employees’ proactive behavior.*

### 2.3. The Moderating Role of AI Literacy

According to the Conservation of Resources (COR) theory, individuals adopt strategies to conserve or replenish valued resources when facing external demands or potential losses (Hobfoll & Shirom, 2000). Within the context of employee–AI collaboration, AI literacy, defined as an employee’s ability to understand, navigate, and effectively apply AI technologies, shapes how effectively individuals can utilize AI tools to reduce workload (Lintner, 2024).

Employees with higher AI literacy levels are typically more skilled and confident in operating intelligent systems, enabling them to integrate AI tools more seamlessly into their workflows. This proficiency allows them to offload repetitive tasks, streamline decision-making, and reduce cognitive strain, leading to greater perceived workload reduction (Cardon et al., 2023; Hornberger et al., 2023). These individuals are also more likely to engage with AI in an adaptive, trust-based manner, which further enhances the benefits of AI-enabled task automation (Ueno et al., 2022).

In contrast, employees with lower AI literacy often lack the technical ability or confidence needed to effectively use AI tools. As a result, they may underutilize available technologies or engage with them inefficiently, limiting workload relief and sustaining resource depletion (Ng et al., 2021). Additionally, uncertainty or skepticism toward intelligent systems can inhibit full engagement, thereby dampening the potential resource-conserving effects of AI collaboration (Su et al., 2023; Vardanyan, 2022).

Taken together, these dynamics suggest that AI literacy strengthens the negative association between employee–AI collaboration and workload, meaning that the more AI-literate employees are, the more they benefit from collaboration in terms of workload reduction.

**H5a:**  *AI literacy moderates the effect of employee–AI collaboration on workload.*

Further, given that AI literacy moderates the effect of employee–AI collaboration on workload, which in turn mediates the relationship between employee–AI collaboration and proactive behavior, we posit that AI literacy also moderates the mediating effect of workload. Specifically, employees with higher AI literacy are more capable of understanding and effectively utilizing AI tools, enabling them to collaborate with AI more efficiently (S.-C. Kong et al., 2025). This facilitates task delegation and process optimization, thereby reducing perceived workload. As a result, these employees conserve more cognitive and emotional resources, which fosters proactive behaviors such as taking initiative, acquiring new skills, or engaging in team collaboration (Pinski & Benlian, 2024; Heyder & Posegga, 2021; Perchik et al., 2023).

In contrast, employees with lower AI literacy may struggle to comprehend the functionality and workflows of AI systems, even when such tools are available. This can lead to increased operational burden and psychological strain, potentially triggering rejection or resistance toward AI integration (Hobfoll & Shirom, 2000; Li et al., 2023). In such cases, AI collaboration may fail to reduce workload, or may even exacerbate it, thereby hindering the emergence of proactive behavior (Reverberi et al., 2022). Based on this reasoning, we propose the following hypothesis:

**H5b:**  *AI literacy moderates the mediating role of workload in the relationship between employee–AI collaboration and proactive behavior.*

**Figure 1:**
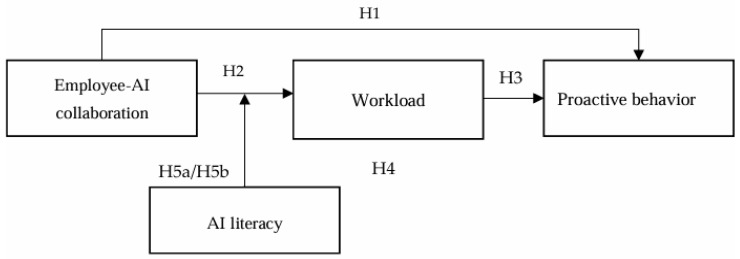
Research Model.

The research model is illustrated in Figure 1.

## 3. Methodology

### 3.1. Procedure and Sample

This study employed a structured questionnaire survey to empirically examine the effects of employee–AI collaboration on employees’ proactive behavior, and to explore the mediating role of workload and the moderating effect of AI literacy. Given the widespread application of employee–AI collaboration across various industries and positions, this study selected enterprises with AI technology applications across multiple industries as research subjects to enhance the external validity of the findings.

The targeted sectors included manufacturing, finance, retail, and internet technology—industries in which employee–AI collaboration is widely practiced and data availability is robust. The sample was drawn from a database of companies that have implemented AI technologies in their operations in China. A total of 30 companies were selected, ensuring diversity in terms of industry and company size.

Within each company, participants were recruited based on organizational size and structural complexity. On average, 15 employees were surveyed per firm, covering a range of positions including frontline staff, technical specialists, and middle-level managers. The proportion of each role reflected the internal workforce composition of the respective organizations. This sampling strategy enabled a comprehensive assessment of how employees across hierarchical levels perceive workload and engage in proactive behaviors in AI-integrated work environments.

Stratified sampling was employed to ensure diversity and representativeness across industries and employee roles. Initial contact was established with management at each participating enterprise to clarify research objectives and secure organizational support.

Within the firms, priority was given to employees who actively interacted with AI tools, intelligent systems, or automation technologies, ensuring the data reflected authentic employee–AI collaboration experiences. Questionnaires were distributed in both paper and digital formats to improve response rates. Prior to distribution, the research team offered a standardized briefing to participants, emphasizing the anonymity and scientific purpose of the study, thereby reducing potential response biases.

Furthermore, to reduce common method bias, a time-lagged design was employed, with the survey being divided into two stages. The first stage, which measured employee–AI collaboration and AI literacy, was conducted in late October 2024, while the second stage, which measured workload and proactive behavior, was conducted in early November 2024, with a two-week interval to effectively minimize biases from collecting data at the same time.

A total of 450 questionnaires were distributed, with 412 valid questionnaires returned, yielding an effective response rate of 91.6%. The sample consisted of 52.4% males and 47.6% females. Regarding age distribution, 21.8% were 25 years old or younger, 48.3% were between 26 and 35 years old, 21.4% were between 36 and 45 years old, and 8.5% were 46 years old or older. In terms of educational background, 76.2% held a bachelor’s degree or higher, 18.7% held a diploma, and 5.1% had a high school education or below. In terms of industry, 28.9% worked in manufacturing, 26.7% in internet technology, 22.5% in finance, and 21.9% in retail. Additionally, 46.8% of the sample consisted of frontline employees, 31.6% were technical staff, and 21.6% were middle managers. The diversity and broad representation of the sample provide strong support for the model testing and generalization of the research conclusions.

### 3.2. Measurement

The variables in this study were measured using established scales from both domestic and international sources. For foreign-language instruments, a strict translation–back-translation process was employed to ensure the accuracy and integrity of the translations. Beyond this standard procedure, we took additional steps to adapt the scales to the Chinese cultural and contextual context. Specifically, two doctoral students, who were highly familiar with both the academic content and the cultural nuances of the target population, carefully reviewed the translated scales. They provided detailed feedback on whether the wording, concepts, and constructs were appropriate for the Chinese context. This process included evaluating the relevance of each item in relation to Chinese cultural norms and ensuring that the items’ meanings were preserved without distortion. Furthermore, adjustments were made where necessary to ensure the scales’ applicability and validity in the Chinese setting. All questions were measured using a 5-point Likert scale, where 1 represented “strongly disagree” and 5 represented “strongly agree”.

The AI literacy variable was measured using the scale developed by B. Wang et al. (2023), with a Cronbach’s alpha of 0.91. A sample item is “I can distinguish between smart devices and non-smart devices”. The employee–AI collaboration variable was measured using the scale developed by H. Kong et al. (2023), with a Cronbach’s alpha of 0.92. A sample item is “AI participates in my decision-making process”. The workload variable was measured using the scale developed by Pickup et al. (2005), with a Cronbach’s alpha of 0.89. A sample item is “How often does your job require you to work very fast?” The proactive behavior variable was measured using the scale developed by Bjørkelo et al. (2010), with a Cronbach’s alpha of 0.84. A sample item is “At work, I would come up with new ideas for completing core tasks”. Additionally, demographic variables such as age and gender were controlled for. Age was measured by an open-ended question, where respondents directly filled in their age, and gender was measured by a multiple-choice question where respondents selected their gender. The complete list of items used for each variable is provided in Appendix A.

### 3.3. Analysis Strategy

Structural Equation Modeling (SEM) was used to examine the hypothesized relationships among employee–AI collaboration, workload, AI literacy, and proactive behavior. Age and gender were included as control variables to account for potential demographic influences and reduce omitted variable bias in the model estimation.

To test the moderation effect of AI literacy, interaction terms between employee–AI collaboration and AI literacy were incorporated into the SEM framework. For the moderated mediation analysis, we applied the PROCESS macro, which enabled simultaneous testing of both indirect (mediation) and conditional (moderated) effects. This approach allowed us to investigate whether the mediating role of workload varies depending on employees’ levels of AI literacy (Hayes, 2017).

These analytical techniques ensure that we thoroughly examine the relationships and interactions among all the variables in our model and provide robust support for our research hypotheses.

## 4. Results

### 4.1. Common Method Bias Test

Firstly, Harman’s single-factor test was used to examine common method bias. All items for the variables were subjected to exploratory factor analysis without rotation. The results showed that the first principal component had an eigenvalue greater than 1, explaining 10.13% of the variance, which did not exceed the 40% critical value. This suggests that common method bias is not a serious issue in this study.

Additionally, given that Harman’s test may lack sensitivity, this study also incorporated an error variable factor based on a five-factor model. The comparison of this model with the five-factor model showed that the changes in the indicators were minimal (CFI = 0.013, ∆TLI = 0.009, ∆RMSEA = 0.008). This provides additional evidence that common method variance does not pose a serious threat to the validity of the results.

### 4.2. Descriptive Statistics Analysis

Table 1 presents the means, standard deviations, and bivariate correlations among the main study variables. Employee–AI collaboration is significantly correlated with workload (r = −0.21, *p* < 0.01), AI literacy (r = 0.27, *p* < 0.01), and proactive behavior (r = 0.28, *p* < 0.01). Workload is significantly negatively correlated with proactive behavior (r = −0.27, *p* < 0.01). These patterns provide preliminary support for the proposed relationships, particularly regarding the associations among employee–AI collaboration, workload, and proactive behavior.

### 4.3. Confirmatory Factor Analysis

This study used Mplus 7.4 for confirmatory factor analysis. First, a four-factor model (baseline model) was constructed, followed by three-factor, two-factor, and one-factor models. The fit statistics for each model are summarized in Table 2.

The four-factor model demonstrated superior fit relative to the alternative models (χ^2^/df = 1.21, RMSEA = 0.05, CFI = 0.98, TLI = 0.97), supporting the distinctiveness of the four latent variables. These results indicate that the measurement model shows good discriminant validity and is appropriate for further analysis.

### 4.4. Hypothesis Testing

**Figure 2:**
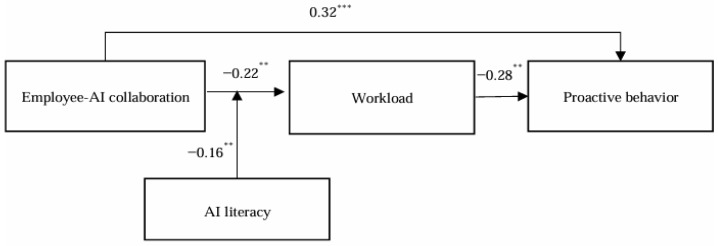
The result of hypothesis test. (** *p* < 0.01. *** *p* < 0.001).

This study used Mplus 7.4 to construct a structural equation model to test the hypotheses. The path coefficients and their significance are shown in Figure 2.

#### 4.4.1. Direct Effect Test

As shown in Figure 2, employee–AI collaboration significantly positively influences proactive behavior (B = 0.32, *p* < 0.001), supporting H1. In addition, employee–AI collaboration significantly negatively impacts workload (B = −0.22, *p* < 0.001), and workload significantly negatively influences proactive behavior (B = −0.28, *p* < 0.001), thus supporting H2 and H3.

#### 4.4.2. Mediation Effect Test

To examine the mediating role of workload between employee–AI collaboration and proactive behavior, the study used the Bootstrap method (5000 resamples) recommended by Preacher et al. (2007). The results show that the mediating effect of workload between employee–AI collaboration and proactive behavior is 0.25, and the confidence interval of the indirect effect at the 95% level is [0.12, 0.32], which does not include zero. This result confirms that the mediating effect of workload is statistically significant, thus supporting H4.

#### 4.4.3. Moderation Effect Test

To examine the moderating effect of AI literacy on the relationship between employee–AI collaboration and workload (H3), we first performed data preprocessing. Specifically, the continuous predictor variables, employee–AI collaboration and AI literacy, were mean-centered prior to analysis. This centering was performed to reduce multicollinearity between the main effects and the interaction term, and to improve the interpretability of the regression coefficients. After centering the continuous predictors, an interaction term (employee–AI collaboration × AI literacy) was computed as the product of their centered values. This interaction was tested within the SEM framework, controlling for age and gender. As shown in Figure 2, the interaction term had a significant negative effect on workload (B = −0.16, *p* < 0.01), indicating that AI literacy moderates the effect of employee–AI collaboration on workload, thus supporting H5a.

**Figure 3:**
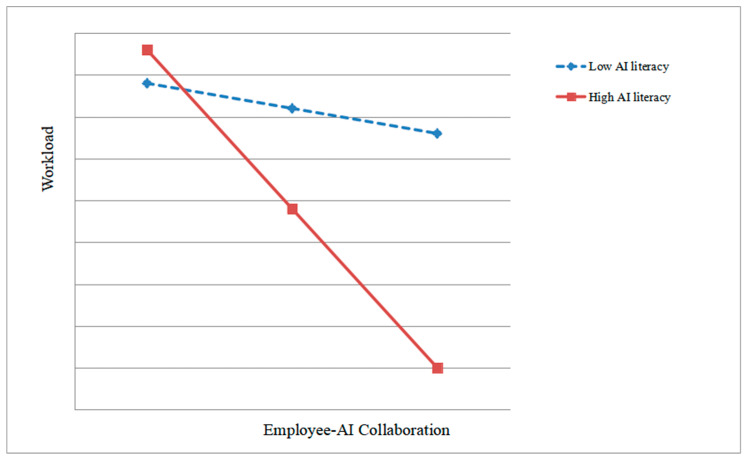
The moderating effect of AI literacy on the relationship between employee–AI collaboration and workload.

To further interpret this interaction, a simple slope analysis was conducted. As shown in Figure 3, the negative relationship between employee–AI collaboration and workload was stronger for employees with high AI literacy and weaker for those with low AI literacy. This suggests that employees with greater AI literacy are better able to leverage AI tools to reduce workload.

#### 4.4.4. Moderated Mediation Effect Test

To examine H4, a moderated mediation analysis was conducted using a bootstrap procedure with 5000 resamples. Table 3 reports the conditional indirect effects of employee–AI collaboration on proactive behavior via workload at different levels of AI literacy.

When AI literacy is low, the indirect effect of employee–AI collaboration on proactive behavior via workload is 0.22, with a 95% confidence interval of [0.12, 0.31], which does not include zero—indicating a statistically significant mediation effect. However, when AI literacy is high, the indirect effect is only 0.01, with a 95% confidence interval of [−0.06, 0.32], whose confidence interval includes zero, indicating non-significance. The difference between these two conditional indirect effects is statistically significant, confirming the moderating effect of AI literacy on the strength of the mediation.

Furthermore, although the moderating effect of AI literacy was statistically supported, the direction of the observed pattern did not fully align with the expectations articulated in H5b. A closer examination of Table 3 reveals an important nuance. Specifically, while the conditional indirect effect is stronger and statistically significant in the low AI literacy group (β = 0.22, 95% CI [0.12, 0.31]), it is weaker and not significant in the high AI literacy group (β = 0.01, 95% CI [−0.06, 0.32]). Notably, the confidence interval in the high literacy group is substantially wider than that in the low literacy group.

This suggests an important theoretical nuance: although the average indirect effect is weaker for high-AI-literacy employees, the dispersion is much greater, pointing to a higher degree of behavioral heterogeneity. In other words, while some high-literacy employees may exhibit elevated proactivity, others may not—possibly due to varying task goals, work identities, or value orientations. These individuals are typically more autonomous, more capable of defining their work priorities, and more sensitive to the contextual meaning of AI-supported collaboration. Such diversity may dilute the observable indirect effect, but it also highlights a latent moderating structure, possibly involving third-order interactions (e.g., personal values, job design, organizational culture).

In contrast, low-AI-literacy employees tend to respond more uniformly to AI collaboration, relying heavily on AI’s workload-reducing function to unlock proactive behaviors. Their responses are more concentrated, more predictable, but also more dependent on external system features than on internal agency. This divergence between the two groups points to a deeper implication: AI literacy not only alters mean effects, but also reshapes the variability and complexity of employee behavioral responses—a perspective worthy of further investigation in future studies.

## 5. Discussion

### 5.1. Theoretical Contributions

This study advances the theoretical understanding of employee–AI collaboration by revealing how such collaboration promotes proactive behavior through workload reduction. While previous research has primarily focused on task performance, efficiency gains, or stress mitigation (Li et al., 2024b; Marvi et al., 2025), we shift the emphasis toward a less-explored mechanism: how AI-enabled collaboration shapes discretionary behaviors such as innovation, process improvement, and change initiation—critical elements of long-term organizational adaptability (Bjørkelo et al., 2010). Grounded in Conservation of Resources (COR) theory, our findings suggest that AI collaboration reallocates employees’ cognitive and psychological resources by relieving workload, thereby fostering conditions conducive to proactive engagement (Hobfoll & Shirom, 2000). Rather than merely reducing strain, AI facilitates a gain cycle in which liberated resources are invested in higher-order, discretionary efforts that benefit both individual development and organizational innovation.

In addition, we clarify a key theoretical pathway by establishing the mediating role of workload. In contrast to prior studies that treated workload reduction as an end in itself, our research identifies it as a core mechanism linking AI use with proactive behavior (Li et al., 2025). This perspective contributes a more nuanced understanding of how technological integration reshapes employee conduct—not merely by enhancing efficiency, but by reallocating internal resources toward intrinsically motivated behavior (Kosch et al., 2023).

Furthermore, this study reveals a dual-layered moderating role of AI literacy in shaping the outcomes of employee–AI collaboration. Consistent with H3, employees with higher AI literacy are better equipped to integrate intelligent systems into their workflows, enabling greater workload reduction through efficient task automation and cognitive offloading (Pinski & Benlian, 2023, 2024).

The moderated mediation analysis (H4), however, yields more nuanced insights. Although the indirect effect of AI collaboration on proactive behavior through workload is statistically stronger among low-AI-literacy employees, a closer inspection of the confidence intervals reveals a much wider distribution in the high-literacy group. This indicates substantial heterogeneity in how high-literacy employees respond to workload changes—some exhibit enhanced proactivity, while others do not. Such variability likely stems from individual differences in task orientation, motivational structures, and autonomy in engaging with AI-supported processes (Bankins et al., 2024; Zhao et al., 2025). In contrast, low-AI-literacy employees tend to rely more uniformly on the workload-reducing function of AI, leading to a more consistent behavioral response (Basri, 2024).

These findings enrich theoretical understanding in several ways. First, they suggest that AI literacy not only moderates the strength of AI collaboration’s impact, but also introduces variation in behavioral responses through cognitive and motivational pathways—an area underexplored in the current human–AI interaction literature. Second, the asymmetry observed between high- and low-literacy groups aligns with resource-based views and self-determination theory, which highlight how internalized goals and competence perception drive the willingness to reinvest saved resources. High-literacy employees, often characterized by greater autonomy and developmental aspirations, may engage with AI in highly differentiated ways, depending on their personal goals, strategic orientation, and prior experience. Third, by showing that technological competence amplifies not only capabilities but also divergence, this study extends the conceptualization of AI literacy from a unidimensional “enabler” to a multifaceted differentiator—shaping not only whether but also how proactive behavior is expressed. Recognizing this complexity calls for future theoretical models to move beyond linear assumptions and account for nested heterogeneity in AI-mediated behavioral processes (Woolley, 2024; Cu et al., 2023; Deci et al., 2017; Xu & Lu, 2022).

Finally, this study adopts a more balanced theoretical stance by acknowledging that AI integration does not uniformly enhance proactive behavior. Ethical concerns, displacement anxiety, and resistance to technological change may offset the anticipated benefits of AI. For example, workload reduction could be interpreted by some employees as a threat to their job security, diminishing their willingness to engage proactively. Future research should account for these countervailing forces to develop a more comprehensive understanding of the behavioral dynamics surrounding AI adoption in the workplace.

### 5.2. Practical Contributions

The findings of this study provide several actionable implications for organizations seeking to enhance employee proactivity through AI integration. First, employee–AI collaboration was found to significantly reduce workload, thereby enabling employees to reallocate cognitive and emotional resources to more innovative and value-added activities. To realize this potential, managers should introduce AI tools that target routine, high-volume tasks such as customer queries or data preprocessing, thereby freeing up capacity for problem-solving and initiative. Examples include AI-powered chatbots and automated analytics dashboards.

Second, the mediating role of workload underscores the importance of work redesign. Organizations can implement AI-enabled workflow systems to streamline mundane activities like scheduling and report generation. In parallel, flexible arrangements such as remote work or adjustable hours can further alleviate psychological strain and enhance cognitive engagement. These interventions not only improve productivity but also create favorable conditions for proactive behaviors, including idea generation and process optimization.

Third, the differential impact of AI literacy suggests the need for stratified support strategies. For employees with lower AI literacy, clearer behavioral benefits can be realized through intuitive interfaces, step-by-step tutorials, and gradual onboarding. These tools can reduce psychological resistance and facilitate faster learning, thereby unlocking proactive potential. In contrast, high-literacy employees demonstrate greater behavioral heterogeneity. For this group, empowerment strategies such as co-design opportunities, advanced scenario workshops, and experimental pilot programs can be more effective in stimulating discretionary effort and innovation. Cross-level mentoring programs, where AI-competent staff assist others, can also bridge skill gaps and promote shared learning.

Finally, promoting AI literacy at an organizational level is essential. By simplifying tool interfaces, fostering a culture of experimentation, and embedding peer support systems, firms can reduce adoption barriers, raise employee engagement, and maximize the long-term strategic value of human–AI collaboration.

### 5.3. Research Limitations and Future Research Directions

This study has several limitations that open avenues for future inquiry. First, although our moderated mediation analysis confirmed a significant effect among low-AI-literacy employees, the expected stronger effect in the high-literacy group was not supported. However, the broader confidence interval observed in the high-literacy group suggests substantial within-group heterogeneity. This indicates that highly AI-literate employees do not respond uniformly to workload relief—possibly due to differences in autonomy, motivational orientation, or role expectations. To better clarify the boundary conditions under which AI literacy promotes proactivity, future studies may introduce third-order moderators, such as job design, goal orientation, or organizational climate, to clarify when and for whom AI literacy facilitates proactive behavior.

Second, this study treated AI literacy as a unidimensional construct. In practice, however, it likely comprises multiple dimensions such as cognitive competence, emotional readiness, and ethical awareness, each of which may interact differently with AI systems. Future research should disaggregate AI literacy into its key components to reveal more nuanced interaction patterns and support the development of tailored training and system deployment strategies.

Third, while this study focused on the positive outcomes of AI collaboration, such as resource conservation and behavioral activation, it may have overlooked potential drawbacks including technostress, resistance, or perceived loss of autonomy. A more critical perspective is needed to explore how concerns over fairness, job security, or identity threat might undermine the expected benefits of AI integration.

Lastly, the data were collected from Chinese organizations with relatively advanced AI adoption, which may limit the generalizability of the findings. Future studies could employ cross-cultural or cross-industry comparative designs to test the model’s applicability under different levels of digital maturity, technological infrastructure, and cultural attitudes toward AI.

Together, these directions highlight the need for more context-sensitive and psychologically grounded research to better understand how AI systems reshape employee behavior, individual agency, and organizational adaptation.

## 6. Conclusions

This study aimed to explore the mechanism by which employee–AI collaboration influences employees’ proactive behavior, examining the mediating role of workload and the moderating role of AI literacy. The findings demonstrate that employee–AI collaboration significantly promotes proactive behavior by reducing employees’ workload. Specifically, employee–AI collaboration not only enhances work efficiency but also alleviates employees’ cognitive burden and psychological stress, thereby encouraging proactive behaviors such as actively seeking innovation and improving work processes.

Additionally, the study found that workload mediates the relationship between employee–AI collaboration and employees’ proactive behavior. By reducing workload, employee–AI collaboration effectively frees up employees’ cognitive resources and time, enabling them to dedicate more energy to proactively undertaking tasks beyond their formal responsibilities. This finding further reinforces the critical role of workload in employee behavior research, particularly during digital transformation, where optimizing workload can effectively boost employee proactivity and innovation.

Importantly, the effects of AI collaboration differ depending on employees’ levels of AI literacy. Consistent with H3, employees with higher AI literacy benefited more from AI in terms of workload reduction, as they were more capable of integrating intelligent systems into their daily routines. However, moderated mediation analysis (H4) revealed a more nuanced pattern: while the indirect effect of AI collaboration on proactive behavior via workload was statistically stronger among low-AI-literacy employees, the broader confidence interval in the high-literacy group indicates greater within-group heterogeneity. This suggests that high-AI-literacy employees may exhibit more diverse behavioral responses to workload reduction—shaped by factors such as autonomy, goal orientation, or task identity—whereas low-literacy employees tend to respond more consistently and predictably, relying more heavily on the relief function of AI.

These findings underscore the importance of individual differences in digital work environments, highlighting that businesses should provide appropriate training and support based on employees’ AI literacy levels to ensure technology’s optimal effectiveness and foster proactive employee behaviors.

## Figures and Tables

**Table 1:** Means, standard deviations, correlations, and reliability among study variables.

Variable	Mean	SD	1	2	3	4	5	6	7
1 Gender	1.34	0.56	1						
2 Age	27.34	2.56	0.12	1					
3 AI Literacy	4.34	1.22	0.21 *	0.31 **	1				
4 Employee–AI Collaboration	4.45	1.59	0.13	0.23 *	0.27 **	1			
5 Workload	5.01	1.51	0.31 **	0.23 *	−0.14 *	−0.21 **	1		
6 Proactive Behavior	4.45	1.32	0.22 *	0.21 *	0.22 *	0.28 **	−0.27 **	1	

Note: * *p* < 0.05, ** *p* < 0.01.

**Table 2:** Confirmatory factor analysis results.

Model	χ^2^/df	CFI	TLI	RMSEA
Four-factor model	1.21	0.98	0.97	0.05
Three-factor model (EAC + WL, PB, AL)	7.34	0.82	0.81	0.16
Two-factor model (EAC + WL + PB, AL)	13.44	0.63	0.72	0.20
Single-factor model (EAC + WL + PB + AL)	16.89	0.49	0.44	0.29

**Table 3:** Moderated Mediation Effect Test Results.

Moderator Variable	Effect	SE	Lower Limit of 95% Confidence Interval	Higher Limit of 95% Confidence Interval
Mean − 1SD	0.22	0.03	0.12	0.31
Mean + 1SD	0.01	0.36	−0.06	0.32

## Data Availability

The data that support the findings of this study are available on request from the first author. The data are not publicly available due to privacy or ethical restrictions.

## Appendix A

Items of Employee–AI collaboration
  1. AI participates in my decision-making process.
  2. AI participates in my prediction process.
  3. AI participates in my problem-solving process.
  4. AI participates in my information identification and evaluation process.
  5. AI participates in my problems, opportunities, or risk recognition process.
Items of Workload
  1. How often does your job require you to work very fast?
  2. How often does your job require you to work very hard?
  3. How often does your job leave you with little time to get things done?
  4. How often is there a great deal to be done?
  5. How often do you have to do more work than you can do well?
Items of Proactive behavior
  1. At work, I would come up with new ideas for completing core tasks.
  2. I would actively seek out ways to improve how my work is done.
  3. I would initiate changes to make my job more efficient or effective.
  4. I would look for opportunities to take on responsibilities beyond my regular duties.
Items of AI literacy
  1. I can distinguish between smart devices and non-smart devices.
  2. I know how AI technology can support work tasks and business processes.
  3. I can identify the AI technology used in the tools and platforms I use at work.
  4. I can skillfully use AI applications or products to support my work and improve performance.
  5. It is usually hard for me to learn to use a new AI application or product. (reverse scored)
  6. I use AI applications or products to enhance work efficiency and effectiveness.
  7. I can evaluate the capabilities and limitations of an AI application or product after using it for a while.
  8. I can choose proper solutions from various AI-driven tools or platforms available at work.
  9. I can select the most appropriate AI application or product for specific tasks in my job.
  10. I always comply with ethical principles when using AI applications or products in my work.
  11. I am always aware of privacy and information security issues when using AI applications or products.
  12. I am always alert to the potential misuse or abuse of AI technology in the workplace.
